# Design and Performance Testing of a Novel Three-Dimensional Elliptical Vibration Turning Device

**DOI:** 10.3390/mi8100305

**Published:** 2017-10-12

**Authors:** Jieqiong Lin, Jinguo Han, Mingming Lu, Jiakang Zhou, Yan Gu, Xian Jing, Da Feng

**Affiliations:** Key Laboratory of Micro-Nano and Ultra-precision Manufacturing of Jilin Province, School of Mechatronic Engineering, Changchun University of Technology, Changchun 130012, China; linjieqiong@ccut.edu.cn (J.L.); hankeyee@163.com (J.H.); zhoujiakang07@163.com (J.Z.); guyan@ccut.edu.cn (Y.G.); zzxb_jx@126.com (X.J.); fengda1994@126.com (D.F.)

**Keywords:** elliptical vibration cutting, three-dimensional elliptical trajectory, flexure hinge, compliant mechanism

## Abstract

A novel three-dimensional (3D) elliptical vibration turning device which is on the basis of the leaf-spring-flexure-hinges-based (LSFH-based) double parallel four-bar linkages (DPFLMs) has been proposed. In order to evaluate the performance of the developed 3D elliptical vibration cutting generator (EVCG), the off-line tests were carried out to investigate the stroke, dynamic performance, resolution, tracking accuracy and hysteresis along the three vibration axes. Experimental results indicate that the maximum stroke of three vibration axes can reach up to 26 μm. The working bandwidth can reach up to 1889 Hz. The resolution and hysteresis tests show that the developed 3D EVCG has a good tracking accuracy, relative high resolution and low hysteresis, which is appropriate for micro/nano machining. Kinematical modeling is carried out to investigate the tool vibration trajectory. Experimental results shown that the simulation results agree well with the experimental one, which indicate that the developed 3D EVCG can be used as an option for micro/nano machining.

## 1. Introduction

Freeform optics with complex geometric features have many advantages. In recent years, the requirement of applications for optical parts in aerospace and industrial production has increased year by year [1,2,3]. However, the further application of optical parts is restricted due to high rates of tool wear, low efficiency of ultra-precision machining and high manufacturing costs [4]. For optical elements manufacturing, generally, fast tool servo (FTS) or slow tool servo (STS) is usually adopted [5,6,7]. Compared with traditional precision machining, FTS or STS do bring a lot of benefits especially for freeform optics manufacturing. However, the ability is limited when comes to the more complicated manufacturing requirements. Therefore, elliptical vibration cutting (EVC) is proposed and recognized as one of the promising ways, and its unique intermittent cutting and friction reversal characteristics caused large attention. In order to obtain an ideal machining results, EVC generator (EVCG) always plays a key role in it. Thus, many studies have been carried out trying to achieve an effective design.

The research of EVC apparatus has grown from two dimensional to three dimensional, and resonance to non-resonance. The two dimensional resonant EVC apparatus was first proposed by Shamoto and Moriwaki [8,9], which was actuated by two piezoelectric plates glued to the lateral surfaces and its resonant frequency is 20 kHz. However, this device has strict requirements when it is fixed, otherwise it is difficult to motivate ideal mode of the transducer. Shamoto improved the EVC device with 4 large piezoelectric plates subsequently and developed an ultrasonic elliptical vibration controller to compensate the vibration interfere in two perpendicular directions, then a 3 degree-of-freedom (DoF) ultrasonic vibration tool was developed for sculptured surfaces [10,11]. Li and Zhang proposed an asymmetrical structural model of ultrasonic elliptical vibration transducer which was driven by single actuator with the longitudinal excitation [12]. Kim and Loh proposed an ultrasonic EVC device for micro V-groove machining which was based on Cerniway’s design [13]. Guo and Ehmann developed a novel device which was composed of two bolt-clamped Langevin transducers [14]. The two bolt-clamped Langevin transducers work in the resonant mode of which the frequency are almost same and deliver an elliptical trajectory at this coupled resonant frequency. As is konwn, all these apparatus summarized above belong to the category of ultrasonic vibration cutting. The biggest advantage of resonant EVCG is the large working frequency. However, this is also a weakness of it due to the fixed working frequency. Therefore, non-resonant EVCG is proposed to increase the manufacturing flexibility during machining.

Compared with resonant EVCG, non-resonant EVCG always have a lower frequency, but the working frequency is continuous, which make the processing process more flexible. The largest number of studies focused on 2D non-resonant EVCG. JH Ahn et al. developed a 2D vibration apparatus for micro-machining accuracy improvement which was actuated by two perpendicular piezoelectric actuators (PEAs), a cross-shaped voids are devised in order to remove cross-interference [15]. Cerniway and Negishi proposed a low-frequency and high-frequency EVC devices which can be operated at 200 Hz with vibration amplitudes of 20 μm × 4 μm and 4 kHz with vibration amplitudes of 18 μm × 3 μm, respectively [16,17]. A 2D vibration cutting tool based on elastic hinges which can produce amplitude of 10 μm and maximum frequency of 500 Hz with no phase distortion was proposed by Ho-Sang Kim et al. [18]. R Kurniawan and TJ Ko developed a non-resonant mode transducer tool holder inspired by Guo’s and Kim’s design proposal to fabricate micro-dimple and groove patterns [19]. Zhou et al. proposed a double-frequency EVC apparatus for freeform surface by using two piezoelectric actuators perpendicularly each other which are both based on the leaf type flexure hinge [20]. In order to obtain a compact design with high working bandwidth and low axis coupling, Lin et al. propose a parallel PEA actuated EVCG [21]. Although these studies propose different method for different considerations to improve the machining performance, it is difficult to achieve a perfect result due to the inherent attribute, such as single vibration direction, mutual conflicting between high working bandwidth and large stroke. In order to obtain a more flexible vibration in space, a flexure-based 3D EVCG actuated by four PEAs are developed by Lin [22]. In addition, a three PEAs actuated 3D EVCG is proposed by Zhu [23]. However, the size is very large and the structure is relatively complicated. Then a novel rotary spatial vibration system is proposed by Zhu to impose tertiary motions with multiple DoFs on a rotating spindle for micro/nanomachining [24]. This design uses three PEAs along three perpendicular directions to generate the 3D motion. The stroke is not very high, and the life of PEA is threatened by transverse force results from transverse motion. Moreover, the compound multibeam parallelogram mechanisms (CMPMs) are nonlinear according to the report of [25]. And the non-linear behavior of the system is a common phenomenon due to the initial internal force [26], material non-linear and large deformations [27]. However, for EVCG, the linear elastic material is often adopted and the deformation is only tens of microns which is relatively small. Therefore, it is appropriate to neglect the non-linear behavior of the system.

As discussed above, there still have some problems in the research of all these developed EVC apparatus, especially for 3D EVCG, less researchers pay attention to it. In this paper, a novel 3D EVCG is proposed aiming to obtain a compact structure with relative large stroke and high working bandwidth to offer more options for micro/nano machining. 

## 2. Mechanical Design

### 2.1. Design of 1-Degree-of-Freedom (DoF) Flexure-Based Compliant Mechanism

**Figure 1:**
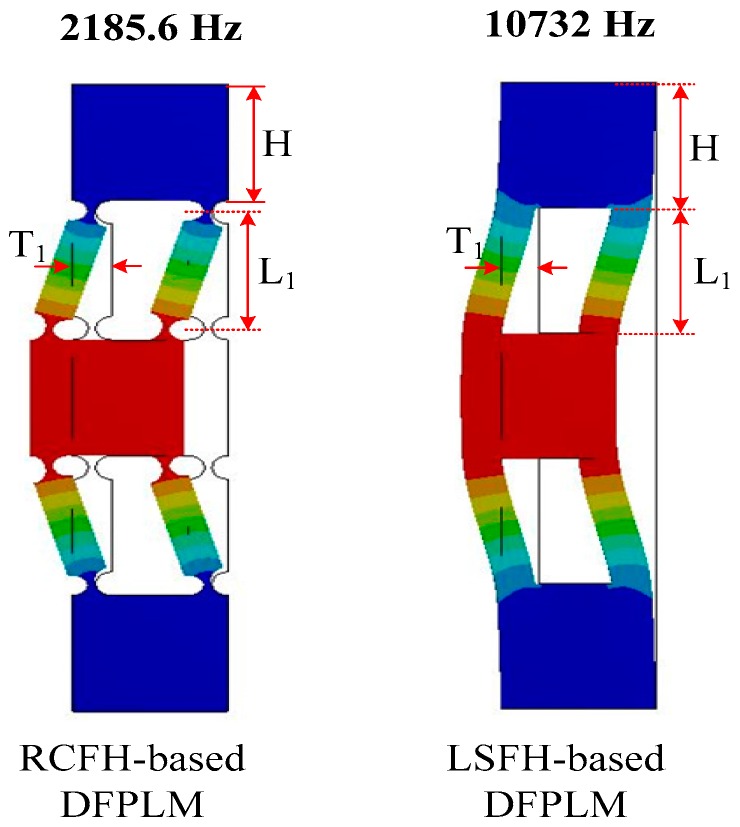
Different configurations of double parallel four-bar linkages (DPFLMs) and its dominant resonant modes.

It is known that applications where the orientation of the end-effector needs to be fixed and the angular motion needs to be eliminated, the parallel four-bar linkages mechanisms (PFLMs) are widely used. According to [28], the most commonly used configurations for PFLMs include right-circular-flexure-hinge-based systems (RCFH-based systems) and leaf-spring-flexure-hinges-based systems (LSFH-based systems). However, the parasitic motions are always exist. In order to avoid the parasitic motions, various double parallel four-bar linkages (DPFLMs) are developed and widely used [20,29,30,31]. In general, the amplifier mechanism is adopted to achieve displacement amplification [32]. In order to investigate the elastic deformation behavior, various methods have been adopted, including finite element method (FEM) [33], Castigliano’s second theorem [32] and pseudorigid body (PRB)-based models [34]. It is easy to carry out the theoretical analysis based on these methods. In this paper, the DPFLM without amplifier mechanism is chosen to achieve a high motion resolution. As shown in Figure 1, the dominant resonant mode of LSFH-based DPFLMs is much larger than the dominant resonant mode of RCFH-based DPFLMs under the same dimensional parameters condition. Besides, the LSFH-based DPFLMs are much easier to be manufactured and it is easy to carry out the theoretical analysis. Without loss of generality, the LSFH-based DPFLMs are adopted as motion guidance.

### 2.2. Structure Design

**Figure 2:**
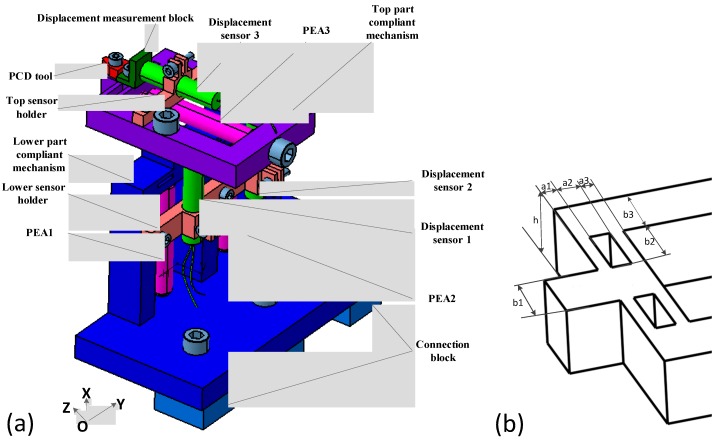
Schematic of 3D elliptical vibration cutting (EVC) device driving by piezoelectric hybrid-actuator. (**a**) Three-deimensional aassemblage diagram, (**b**) Motion partial structure.

In this paper, a PEA-actuated non-resonant 3D EVCG is developed on the basis of three DPFLMs. The mechanical structure is schematically illustrated in Figure 2. As is shown in Figure 2a, two parallel PEAs in vertical direction and one PEA in horizontal direction is adopted to drive the compliant mechanism. The structure of developed apparatus is mainly composed of two parts: the top part and the lower part. The lower part compliant mechanism can be fixed on lathe by the connecting block, and three precision screws are utilized for the PEA preloads. The PEA1 and the PEA2 are fixed on the lower part compliant mechanism, and the PEA3 is fixed on the top part compliant mechanism. For easy analysis, three axes are defined, e.g., the axis along PEA1 is defined as *x*1, axis along PEA2 is defined as *x*2, and axis along PEA3 is defined as *z*1. The structural parameters of the development 3D EVC device are shown in Figure 2b and Table 1. It should be noted that the three PEAs’ motion are approximately independent of each other, there is no transverse motion introduced which is helpful to extend the life of PEAs from shearing effects. In addition, the 3D elliptical vibration can be obtained when three input displacement signals with different phase shifts are adopted. The displacement signal generated by PEA can be acquired by three displacement sensors which are fixed on the apparatus through sensor holders. As feedback signals, these displacement signals can be used to form a closed-loop control. 

## 3. Performance Testing and Discussion

### 3.1. Experimental Setup

**Figure 3:**
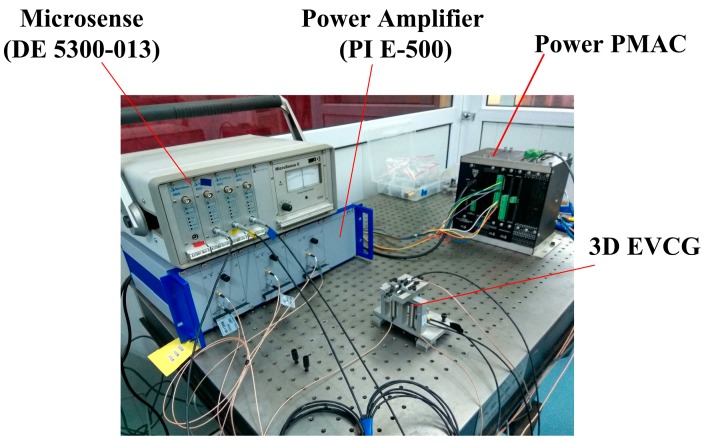
Schematic configuration of experiment setup.

In order to investigate the performance of the developed 3D EVCG, off-line tests were carried out. The experimental setup is illustrated in Figure 3. Three PEAs (40vs12, Harbin Core Tomorrow Science & Technology Co., Ltd., Harbin, China) was chosen to drive the 3D EVCG. The driving signals were amplified by the power amplifier (E500, PI Inc., Athens, TN, USA) and then applied to the PEAs to achieve the vibration. The displacements were measured by capacitive transducers and act as a feedback signals to form a closed-loop control. A power PMAC (Delta Tau Data Systems Inc., Chatsworth, CA, USA) was chosen as the controller. In order to reduce the external disturbances, the off-line tests were carried out on a vibration-isolated air-bearing platform. 

### 3.2. Stroke Test

**Figure 4:**
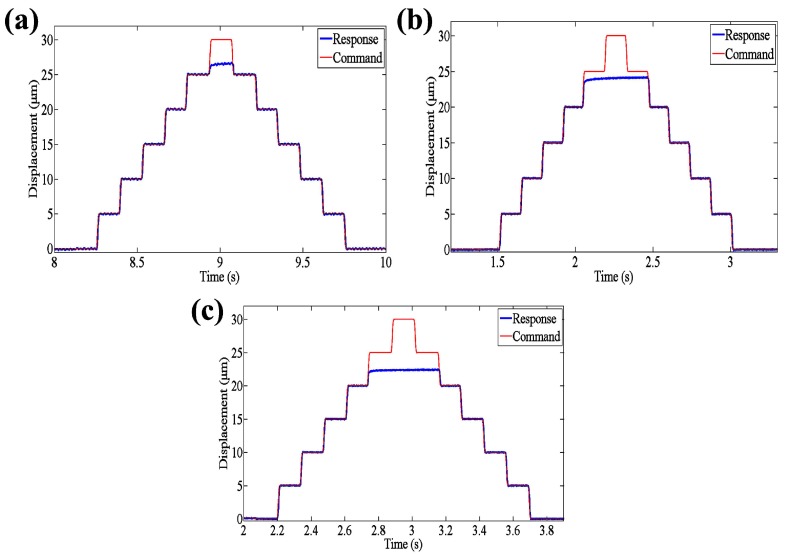
Motion stroke of (**a**) *z*1 axis (**b**) *x*1 axis and (**c**) *x*2 axis.

As is known, the large stroke of EVCG can enhance the machining dimension, which is very important for micro/nano machining, especially for microstructures and freeform surfaces fabrication. Thus, a consecutive step input with maximum displacement 30 μm was adopted as the driving signal for each input end, separately. The results are shown in Figure 4. From Figure 4a, it can be seen that the stroke along *z*1 axis can reach up to 26 μm. And the stroke along *x*1 and *x*2 axes are 24 μm and 22 μm, respectively. Although the structure is symmetry along *xz* plane, the stroke of *x*1 axis and *x*2 axis are different. The reason can be attributed to the manufacturing error. Because the up part compliant mechanism is assembled on the lower part compliant mechanism which is can be seen an external load, thus the stroke is little smaller than that of *z*1 axis. Compared with that in [24], the stroke of the developed 3D EVCG has a lot of improvement.

### 3.3. Dynamic Test

**Figure 5:**
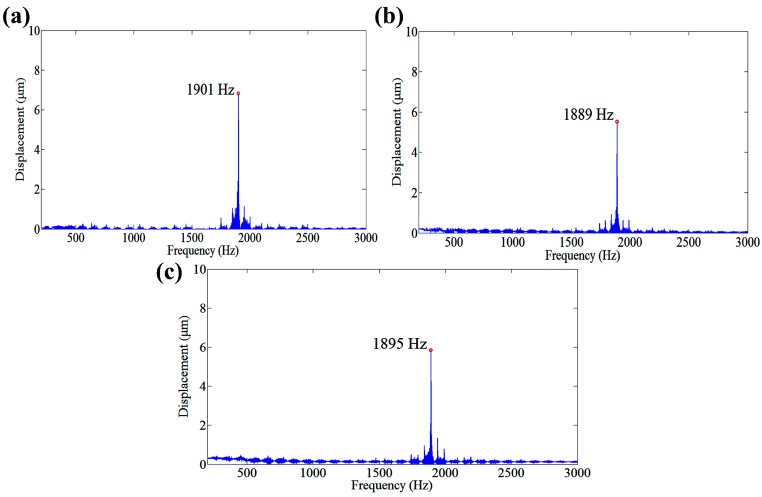
Dynamic response along (**a**) *z*1 axis (**b**) *x*1 axis and (**c**) *x*2 axis.

As shown in Figure 5, the dynamic performance is carried out by using swept excitation method from 100 Hz to 3000 Hz. The output displacements can be obtained by capacitive transducers. And through the fast fourier transformation (FFT), the displacement-frequency response can be deduced by matlab software. The results show that the first natural frequency along *z*1, *x*1 and *x*2 axes are about 1901 Hz, 1889 Hz and 1895 Hz, respectively. The similar first natural frequencies are results from the similar structure. As a machining system, the working bandwidth is restricted by the lowest first natural frequency. Therefore, the first natural of the machining system is 1889 Hz. It is enough for ultra-precision machining.

### 3.4. Resolution Tests and Tracking Accuracy Analysis

**Figure 6:**
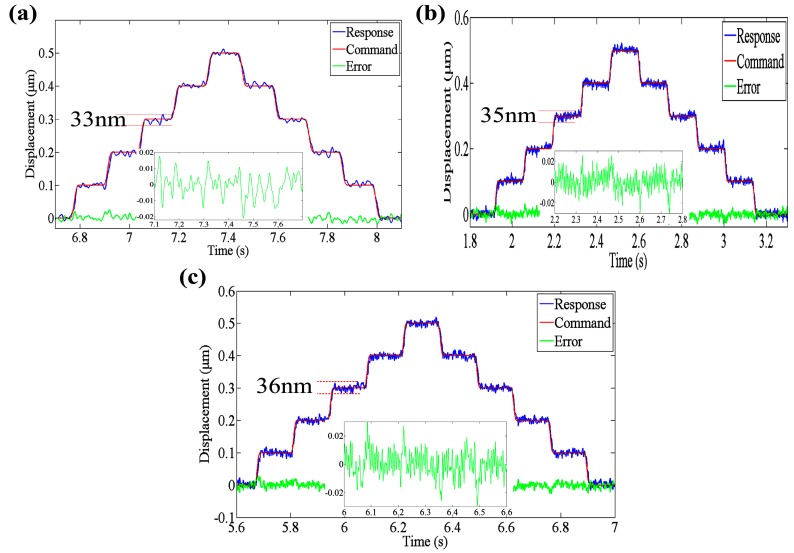
Resolution tests of (**a**) *z*1 axis (**b**) *x*1 axis and (**c**) *x*2 axis.

It is known that the EVC is widely used in freeform and microstructure fabrication. The machining accuracy determined the performance of the machined components. The resolution of the EVCG is very important, because the minimum size of processing characteristics is dependent on the resolution. Stair excitation signals were adopted to investigate the resolutions of developed 3D EVCG. It can be seen from Figure 6, the resolutions of the compliant mechanism along *z*1, *x*1 and *x*2 axes are 33 nm, 35 nm and 36 nm, respectively. Considering the applications of the EVCG [23,24], the resolution can satisfy the freeform and submicron scale microstructures’ fabrication. In addition, according to Figure 6, the tracking errors of three motion axis are all within 0.03 μm, which indicate a good tracking ability of the developed 3D EVCG.

### 3.5. Hysteresis Analysis

**Figure 7:**
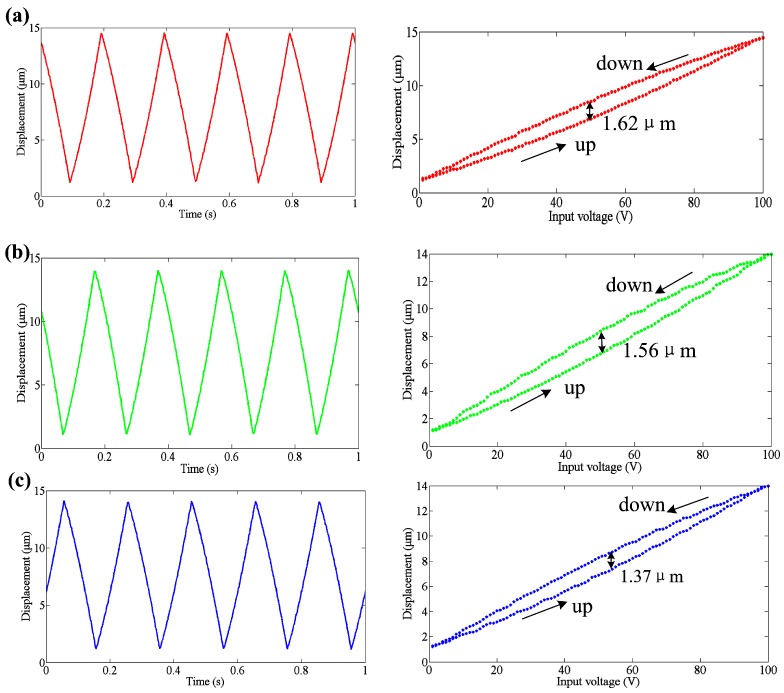
Motion performance (**a**) *z*1 axis (**b**) *x*1 axis and (**c**) *x*2 axis.

The developed 3D EVCG has a hysteresis phenomenon due to the reality of hysteresis of the PEAs. In order to investigate the hysteresis of the developed 3D EVCG, a triangle voltage with 10 V amplitude and 5 Hz frequency was generated by signal generator and applied to the three PEAs, separately. Figure 7 shows the input signals and the corresponding input voltage-displacement responses. The lower curve denotes the expansion of PEA and the upper curve denotes the retraction of PEA. It can be seen that the maximum positioning differences of three motion axes between expansion and retraction are 1.62 μm, 1.56 μm and 1.37 μm, respectively, which is about 11.2%, 11.1% and 9.8% of the displacements under 100 V input voltage. Thus, in our future work, an appropriate control strategy is necessary to reduce the hysteresis phenomenon. 

## 4. Kinematical Modeling and Experimental Validation

### 4.1. Kinematical Modeling

In order to drive the developed 3D EVCG to work, three PEAs are applied with three sinusoidal signals with a certain phase difference as follows:

(1) $$\left\{ \begin{array}{l} x_{1}=A_{1}\sin(\omega_{1}t+\varphi_{1})\\ x_{2}=A_{2}\sin(\omega_{2}t+\varphi_{2})\\ x_{3}=A_{3}\sin(\omega_{3}t+\varphi_{3}) \end{array}\right.$$

where $A_{1},A_{2},A_{3}$ are the input signal amplitude of three PEAs, respectively; $\omega_{1},\omega_{2},\omega_{3}$ are the input signal angular frequency of three PEAs, respectively; $\varphi_{1},\varphi_{2},\varphi_{3}$ are the phase shift of input signals, respectively. 

**Figure 8:**
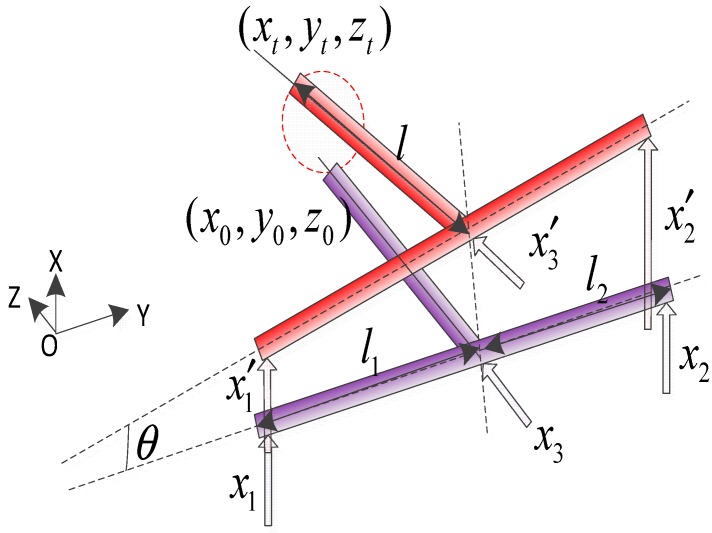
The cutter location calculation model of diamond tool.

In order to illustrate the operational principle of the developed flexure-based 3D EVCG, the cutter location calculation model of diamond tool was established in this paper, as shown in Figure 8.

**Figure 9:**
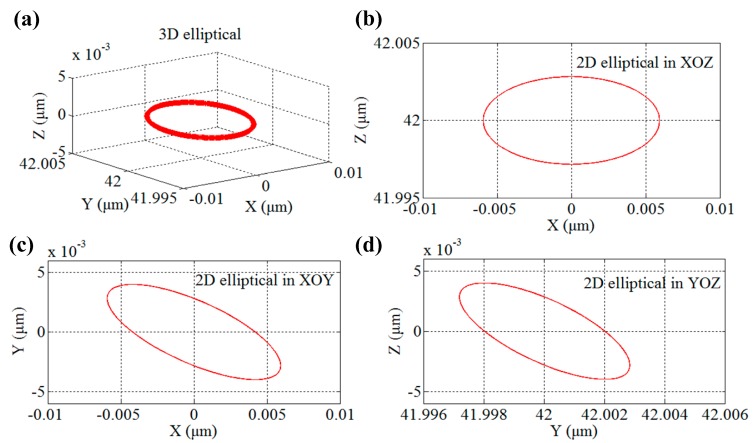
Cutter location numerical simulation of diamond tool in 3D elliptical vibration cutting (EVC). (**a**) Spatial pose. (**b**) Projection in *xoz* plane. (**c**) Projection in *xoy* plane. (**d**) Projection in *yoz* plane.

In Figure 9, the initial position of the diamond tool tip is $\left(x_{0},y_{0},z_{0}\right)$. The three initial displacement driving signals of three PEAs are denoted as $x_{1},x_{2},x_{3}$, respectively. During the operation process, the PEA is expansion and retraction, and the initial signals are transformed into $x_{1}^{\prime },\;x_{2}^{\prime },\;x_{3}^{\prime }$. Due to the phase shift between the driving signal of PEA1 and the PEA2, the top part compliant mechanism will generate an angle θ with the initial position, which can be expressed as:

(2) $$\theta =\arcsin\left(\frac{x_{1}^{\prime }-x_{2}^{\prime }}{l_{1}+l_{2}}\right)$$

where $x_{1}^{\prime },\;x_{2}^{\prime }$ is the current displacements of the PEA1 and the PEA2 in *x* direction, respectively; $l_{1},l_{2}$ is the vertical distance between the two PEAs and diamond tip in *y* direction.

The leaf type flexure hinge was used as the guide elastic element in the top part and lower part compliant mechanism which are designed to be parallel and symmetric structures. The motion trajectory of diamond tool tip $\left(x_{t},y_{t},z_{t}\right)$ can be obtained as follows:

(3) $$\left\{ \begin{array}{l} x_{t}=l\cdot \sin\theta \\ y_{t}=l\cdot \cos\theta +x_{i}^{\prime }+l_{i}\cdot \tan\theta \\ z_{t}=x_{3}^{\prime } \end{array}\right.\begin{array}{cc} & \left(i=1,2\right) \end{array}$$

where *l*_1_ = 20 mm, *l*_2_ = 20 mm, *l* = 42 mm are adopted in this developed 3D EVCG. Taking the simplified connection point between top part compliant mechanism and the lower part compliant mechanism as the original point, and setting the parameters of driving signals of the PEAs as: *A*_1_ = *A*_2_ = *A*_3_ = 4 μm, *ω*_1_ = *ω*_2_ = *ω*_3_ = 100 Hz, *φ*_1_ = 0, *φ*_2_ = π/2, *φ*_3_ = π. The cutter location calculation model can be simulated by using the matlab software, and the trajectory of diamond tool tip can be obtained which is shown in Figure 9. It can be seen from the developed 3D EVCG cutter location trajectory that the projections of diamond tool locus are ellipse in *xy*, *yz* and *xz* planes. In addition, the elliptical trajectory of diamond tool tip can be changed flexibly by adjusting the related parameters.

### 4.2. Experimental Validation

**Figure 10:**
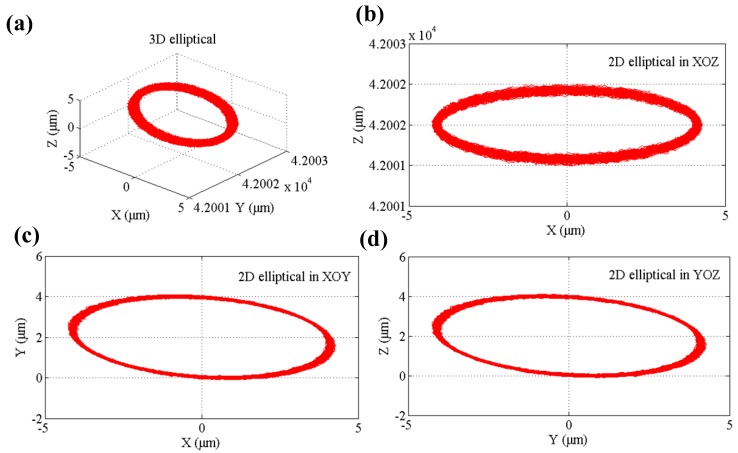
The synthesized tool locus of 3D elliptical vibration cutting (EVC). (**a**) Spatial pose. (**b**) Projection in *xoz* plane. (**c**) Projection in *xoy* plane. (**d**) Projection in *yoz* plane.

In the developed flexure-based 3D EVCG, the probes of the displacement sensor are placed parallel with the PEAs and fixed by the sensor holder. By setting the driving parameters same as the MATLAB simulation in Section 4.1, the output displacements are measured and acquired by the capacitive transducers. In order to validate the efficiency of the kinematical model, the diamond tool tip trajectory is synthesized and shown in Figure 10. As shown in Figure 10, the synthetic diamond tool tip trajectory in experiment is same as the simulation results except the angular error of projection in *xy* and *yz* plane which may be caused by the PEAs’ hysteresis. It should be noted that the formed 3D elliptical locus is smooth and steady, which indicates the good stability of the system.

## 5. Conclusions

In this paper, a novel flexure-based 3D EVC apparatus has been developed, aimed at solving the processing limitations of optical parts with complex geometric features. The developed 3D EVCG is composed of two compliant mechanisms on the basis of LSFH-based DPFLMs. Taking advantage of symmetry design and independent motion of PEAs, there is no transverse motion introduced which is helpful to extend the life of PEAs from shearing effects.

In order to investigate the performances of the developed 3D EVCG, off-line tests were carried out. Results show that the stroke along *z*1, *x*1 and *x*2 axes are 26 μm, 24 μm and 22 μm, respectively. The working bandwidth can reach up to 1889 Hz. The resolutions of the compliant mechanism along *z*1, *x*1 and *x*2 axes are 33 nm, 35 nm and 36 nm, respectively. In addition, the tracking errors of three motion axis are all within 0.03 μm, which indicate a good tracking ability of the developed 3D EVCG. The maximum positioning differences of three motion axes between expansion and retraction are 1.62 μm, 1.56 μm and 1.37 μm, respectively, which is about 11.2%, 11.1% and 9.8% of the displacements under 100 V input voltage. Finally, the cutter location model of diamond tool tip is established based on the driving characteristics of developed apparatus. The simulation results agree well with the experiments’, which indicate that the developed novel flexure-based 3D EVCG has a good performance and it can be used in 3D EVC experiments. In our future work, the developed 3D EVCG will be used to machining experiment to exam the machining performance.

## Figures and Tables

**Table 1:** The structural parameters of the development 3D elliptical vibration cutting (EVC) device.

Parameters	H (mm)	a1 = a3 (mm)	a2 (mm)	b1 = b2 = b3 (mm)
Value	10	3	2	10
